# Acknowledgement to Reviewers of *JPM* in 2018

**DOI:** 10.3390/jpm9010006

**Published:** 2019-01-17

**Authors:** JPM Editorial Office

**Affiliations:** MDPI, St. Alban-Anlage 66, 4052 Basel, Switzerland; jpm@mdpi.com

Rigorous peer-review is the corner-stone of high-quality academic publishing. The editorial team greatly appreciates the reviewers who contributed their knowledge and expertise to the journal’s editorial process over the past 12 months. In 2018, a total of 38 papers were published in the journal, with a median time to first decision of 27.5 days and a median time to publication of 65.5 days. The editors would like to express their sincere gratitude to the following reviewers for their cooperation and dedication in 2018:
Alaimo, AlessandroMather, CareyAlfirevic, AnaMeng, JinhongAntoñanzas, FernandoMills, RachelBien, Stephanie A.Mitchell, Cassie S.Boisvert, François-MichelMoyle, LouiseBonham, Vence L.Naj, AdamBurns, KatieNavab, MohamadButler-browne, GillianNigam, SanjayCastel, VictoriaO’Rourke, Patricia PearlCheng, XiPan, MingguiChmurzynska, AgataPatch, ChristineČipak Gašparović, AnaPerera, MinoliClementi, EmilioPerez, LaurentCodacci-Pisanelli, GiovanniPergament, EugeneCohen, StephaniePersson, CeciliaCrona, DanielPfau, Steven E.Defesche, Joep C.Pyles, Lee A.DeLeon, JoseRadhakrishnan, KavitaDeWald, TracyRagia, GeorgiaDijkstra, JoukeReynolds, Jessica L.Du Preez, IlseSanderson, SaskiaEngvall, JanSandman, LarsEwing, AdamSavoia, CarmineGagliardi, Paolo ArmandoScott, Erick RGallegos, Martha PatriciaSharfaei, SadafGarrison, LouisSharma, AtulGibson, GregSharma, NaveenGlueck, Charles J.Shipman, KateGong, YanSilva, Susana N.Gouin-Thibault, IsabelleSingh, SonalGoyenvalle, AurélieSingla, BhupeshGraber, JoelSkinner, DebraHagenbuch, BrunoStewart, AlexandreHart, StevenTaylor, CaseyHerraez, ElisaToffoli, GiuseppeHiratsuka, MasahiroTolstikov, VladimirHizel, CandanTougeron, DavidHoppu, UllaVafiadaki, ElizabethHult, Kristopher J.Van Putten, MaaikeHvas, Anne-MetteVan Schaik, Ron H.N.Inamura, KentaroVickers, Kasey C.Janowski, MiroslawVilla, ChiaraJirák, DanielVimaleswaran, Karani S.Karnes, Jason H.Wang, XinwenKato, YukioWelch, CarrieKulak, WojciechWilliams, Janet K.Lanfear, DavidWilton, SteveLazzeri, ChiaraWojtkiewicz, JoannaLewis, GladiusWu, AlanLi, YvonneZaiss, Dietmar M.W.López Fernández, LuisZhou, JingyingMaeda, HiroshiZiegelstein, Roy

